# Earwigs from Brazilian caves, with notes on the taxonomic and nomenclatural problems of the Dermaptera (Insecta)

**DOI:** 10.3897/zookeys.713.15118

**Published:** 2017-11-02

**Authors:** Yoshitaka Kamimura, Rodrigo L. Ferreira

**Affiliations:** 1 Department of Biology, Keio University, 4-1-1 Hiyoshi, Yokohama 223-8521, Japan; 2 Center of Studies in Subterranean Biology, Biology Department, Federal University of Lavras, CEP 37200-000 Lavras (MG), Brazil

**Keywords:** bat guano, cave fauna, *Cylindrogaster cavernicola* sp. n., Cylindrogastrinae, dermapteran taxonomy, female genitalia, *Heterolabis*, new synonym, traumatic mating

## Abstract

Based on samples collected during surveys of Brazilian cave fauna, seven earwig species are reported: *Cylindrogaster cavernicola* Kamimura, **sp. n.**, *Cylindrogaster* sp. 1, *Cylindrogaster* sp. 2, *Euborellia janeirensis*, *Euborellia brasiliensis*, *Paralabellula dorsalis*, and *Doru luteipes*, as well as four species identified to the (sub)family level. To date, *C. cavernicola* Kamimura, **sp. n.** has been recorded only from cave habitats (but near entrances), whereas the other four organisms identified at the species level have also been recorded from non-cave habitats. Wings and female genital structures of *Cylindrogaster* spp. (Cylindrogastrinae) are examined for the first time. The genital traits, including the gonapophyses of the 8^th^ abdominal segment shorter than those of the 9^th^ segement, and venation of the hind wings of Cylindrogastrinae correspond to those of the members of Diplatyidae and not to Pygidicranidae. This is the first synopsis of cave-dwelling earwigs of Brazil, one of the most species-rich areas of Dermaptera in the world.

## Introduction

Dermaptera (earwigs) is a polyneopteran insect order with more than 2000 described species from mainly tropical and warm temperate regions (Popham 2000; Grimaldi and Engel 2005; Haas et al. 2012). Although many dermapteran families have circumtropical distributions (Popham 2000), faunal, taxonomic, and ecological studies are scarce for tropical or subtropical species, especially the cave-dwelling species.

Organisms that live in the subterranean environments are frequently classified into three categories (e.g., Souza-Silva et al. 2011): i) trogloxenes - species occasionally found in caves or using the caves as nighttime or daytime shelters; ii) troglophiles – that could complete their whole life cycle inside or outside the caves; and iii) trogolobites species that do not occur in epigean habitats and exhibit behavioral, morphological and physiological specializations for exclusive survival within caves.

Only two troglobitic earwigs have been reported to date: *Anisolabis howarthi* Brindle, 1980 (Anisolabididae) of Hawaiian lava caves and *Anataelia troglobia* Martín and Oromi, 1988 (Pygidicranidae) of a lava cave in La Palma, Canary Islands. These species exhibit the characteristics of true cavernicolous insects, including reduced, apparently non-functional compound eyes, slender appendages, and a less-pigmented integument (Brindle 1980a; Martín and Oromi 1988). The genus *Anataelia* is endemic to the Canary Islands and the other two species, *A. canariensis* Bolivar, 1899 and *A. lavicola* Martín & Oromi, 1988, have well-developed eyes and are found both in subterranean and in epigean habitats (Ashmole and Ashmole 1987; Ashmole et al. 1992).

*Haplodiplatys milloti* (Chopard, 1940) (Haplodiplatyidae), which has been exclusively reported from an entirely dark part of an African cave, has well-developed compound eyes, while the integument is pale and the appendages are slenderer than in related species (Chopard 1940). It is estimated that this species recently adapted to cave life and has experienced minimal morphological changes (Brindle 1980a). Similarly, *Challia phoenix* Anisyutkin & Gorokhov, 1998 (Pygidicranidae sensu stricto) was described based on a single male specimen collected from a cave of Vietnam. Although its relationships to the cave habitat is unclear, according to the descriptions by Anisyutkin and Gorokhov (1998), this species shows no sign of reduction in the compound eye.

Conversely, eyeless earwigs do not necessarily occur in caves. *Anophthalmolabis* spp. (Anisolabididae) (Brindle 1968; Peck 1990) and *Caecolabia gomyi* Brindle, 1975 (Spongiphoridae) (Brindle 1975), are eyeless but usually found from soils. Peck (1990) reported that *Anophthalmolabis leleupi* Brindle, 1968, which is endemic to the Galapagos Islands, also occurs in caves (possibly troglophilic).

Members of the family Arixeniidae are also considered trogrophilic. These species are phoretic on bats (*Cheiromeles torquatus* Horsfield, 1824) or are found on bat guano (Nakata and Maa 1974; Marshall 1977). However, as the bat hosts roost in caves and tree hollows, arixeniids are not categorized as true cavernicolous animals. Nevertheless, they have reduced compound eyes as an adaptation to dark habitats (e.g., Nakata and Maa 1974). Similarly, in members of the Hemimeridae, which are phoretic on African tunnel-living giant rats (*Cricetomys* spp.), eyes are entirely absent (e.g., Nakata and Maa 1974). Morphological and molecular phylogenetic studies showed that both Hemimeridae and Arixeniidae are specialized in-groups of the superfamily Forficuloidea that also includes free-living earwigs of the families Spongiphoridae, Chelisochidae, and Forficulidae (Klass 2001; Haas and Klass 2003; Jarvis et al. 2005; Tworzydlo et al. 2013; Kocarek et al. 2013; Naegle et al. 2016).

Most earwigs found in caves are considered troglophiles that show no apparent specialization for life in dark environments (Chopard 1921, 1924; Brindle 1980a). Examples include *Chelisoches morio* (Fabricius, 1775) (Chelisochidae) and *Schizochelisoches brevipennis* (Borelli, 1923) (Chelisochidae) of Malaysian caves (Chopard 1929; McClure et al. 1967), and *Carcinophora americana* (Palisot de Beauvois, 1817) of Puerto Rican caves (Peck 1974).

As one of the most species-rich areas in the world, approximately 150 species of earwigs have been reported from Brazil (Haas 2012). Nevertheless, except for some rare mentions of Dermaptera in cave environments (Pinto-da-Rocha 1995), no comprehensive review has been reported for cave dermapteran fauna of this country. As an attempt to fill this gap, this is the first synopsis of Brazilian cave-dwelling earwig. Based on the samples collected during surveys of Brazilian cave fauna, we report seven earwig species from cave habitats for the first time. Wing and genital structures are described for some species, based on which we discuss their classification, ecology, and behavior.

## Materials and methods

The 93 dermapteran specimens examined in this study were collected from 2000 to 2015 during surveys of Brazilian cave fauna. All samples were collected manually. Most caves were visited only once in the inventories of subterranean invertebrates conducted in different research projects. Several samples were collected by consulting companies or governmental institutions (ATIVO AMBIENTAL, CARSTE, SPELAYON, or CECAV), for which detailed environmental conditions of the locality are unknown.

All samples were preserved in 70% ethanol after collection, and therefore the body color of some specimens was bleached. To examine wing and genital structures, several adult samples were dried after mounting on cardboard using fish glue. Genitalia were removed from specimens, mounted in Euparal (Waldeck GmbH and Co. KG, Münster, Germany) between two coverslips, and attached to the pin of the respective specimen.

All of the samples examined in this study have been deposited in the Subterranean Invertebrate Collection of Lavras (ISLA), of the Universidade Federal de Lavras (UFLA), Lavras, Brazil, with assignment of sample numbers shown in parentheses below, with the exception of some comparative samples from other depositories.

We follow Engel and Haas (2007) for the suprageneric classification, except for the subfamily Cylindrogastrinae which we considered to belong in Diplatyidae (see below). In addition, Engel et al. (2017) proposed to move the genus *Haplodiplatys*, which is considered the oldest offshoot of the extant Dermaptera (Haas 1995; Hass and Kukalová-Peck 2001; Hass and Klass 2003), from Diplatyidae to Haplodiplatyidae Engel, 2017. We follow this view. The generic classification follows that of Steinmann (1986, 1989a, 1989b, 1990, 1993) unless otherwise noted. The terminologies of Hass and Kukalová-Peck (2001), Klass (2003), and Kamimura (2014) were used for wing, female genital, and male genital structures, respectively.

### Abbreviations

***Male genitalia***

**dp** denticulated pad

**ho** horn

**pm** paramere

**rsc** rectangular sclerite

**tp** toothed plate

**vg** virga

***Female genitalia***

**ap** anal plate

**gl9** gonoplac (=coxal lobe) IX

**gp8** gonapophysis VIII

**gp9** gonapophysis IX

**lp** lateral plate

**sa** spined area

**sp** spermatheca

**tg10** tergum X

***Wing structures***

**AA3** anal anterior 3

**AA4** anal anterior 4

**AP** anal posterior

**BAA1+2** anal anterior 1 + 2 basivenale (anal brace)

**BAA3+4** anal anterior 3 + 4 basivenale

**C** costa

**CuA** cubitus anterior

**CuP** cubitus posterior

**FAJ** anojugal fulcalare

**JA** jugal anterior

***Depositories***

**ISLA** Invertebrados Subterrâneos de Lavras (Subterranean Invertebrates, Lavras) of UFLA

**MM** Manchester Museum, UK

**OMNH** Osaka Museum of Natural History, Japan

**UFLA** Federal University of Lavras, Brazil

**YK** personal collection of Y. K.

## Results and remarks

Apart from *Cylindrogaster*, for which a new species is described, eight organisms were recorded in this study; however, only four were determined to the species level, while four were determined to the (sub)family level.

### Order DERMAPTERA de Geer, 1773

#### Infraorder PROTODERMAPTERA Zacher, 1910

##### Family DIPLATYIDAE Verhoeff, 1902

###### Subfamily CYLINDROGASTRINAE Maccagno, 1929

####### *Cylindrogaster* Stål, 1855

######## Cylindrogaster cavernicola

Taxon classificationAnimaliaDermapteraPygidicranidae

Kamimura, sp. n.

http://zoobank.org/CDD2C2A8-007B-4BF5-BCB7-B2C39D2427AC

Figs 1–7

######### Material examined.

Holotype ♂, ‘Gruta Apertar | da Hora | Jandaíra RN <= Rio Grande do Norte>’, ‘ISLA | 21101’, ‘15.ii.2010 | Ferreira, R.L. leg.’, ‘HOLOTYPE (male) | *Cylindrogaster cavernicola* | sp. n. | Det. Y. Kamimura 2017’.

######### Diagnosis.

*Cylindrogaster cavernicola* sp. n. is a median-sized species with a slender abdomen and simple forceps. This species differs from all other species of *Cylindrogaster* with the combination of the following characters: the well-developed tegmina; pronotum slightly longer than broad; parameres with blunt apices; and short but weakly sinuated virgae.

######### Description.

**Figures 1-11.**
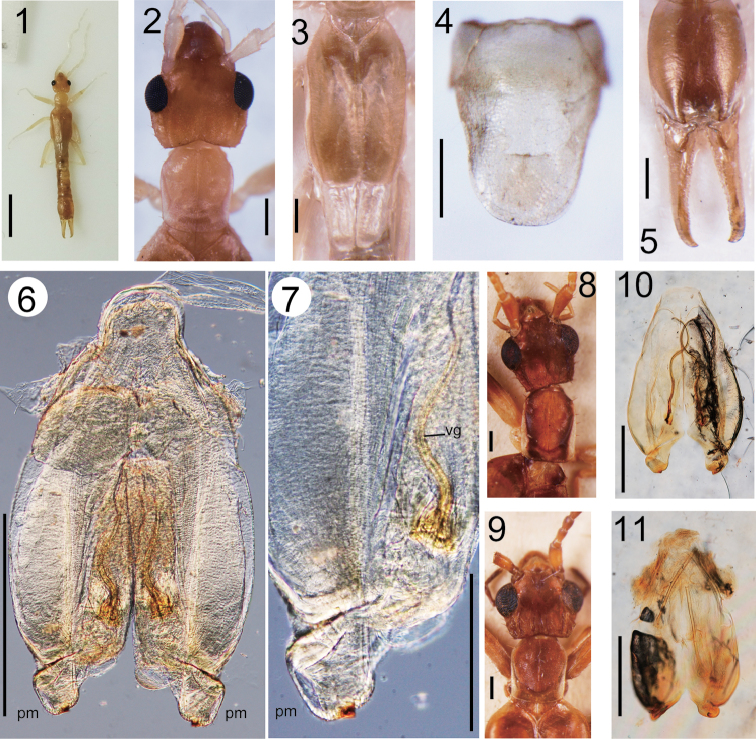
**1-7** *Cylindrogaster cavernicola* Kamimura, sp. n. (male, holotype): habitus (**1**), head and thorax (**2**), tegmina and wings (**3**), penultimate sternite (**4**), ultimate tergite and forceps (**5**), and genitalia (**6, 7**) **8, 10** *Cylindrogaster thoracicus* (MM 3637; collected from Itatiaia, Brazil; det. W. D. Hincks): head and thorax (**8**), and genitalia (**10**) **9, 11***Cylindrogaster gracilis*: head and thorax (**9**: MM 3677; collected from Itatiaia, Brazil; det. W. D. Hincks), and genitalia (**11**: MM 3565; collected from Minas Gerais, Brazil; det. W. D. Hincks). Scale bars 3 mm for Fig. **1**; 0.5 mm for Figs **2-6, 8-11**; 200 ?m for Fig. **7**.

***Male*** (holotype: Fig. 1). Length of body (without forceps): 11 mm. Length of forceps: 1.4 mm. Head width: 1.5 mm. Pronotum width: 1.0 mm. Pronotum length: 1.3 mm.

The body color of the holotype seems to have been bleached by preservation in ethanol. Body color uniformly pale amber but abdomen darker. Body, including forceps, sparsely pubescent.

Head (Fig. 2) slightly longer than broad, widest in the region of the eyes; frons tumid, occiput depressed; transverse and median suture not conspicuous but visible; hind margin strongly emarginated in middle; post-ocular carina well developed, almost straight, running from middle of the internal margin of the eyes to the hind margin of the head; lateral margins of post-ocular region bordered with strong bristles. Antennae broken, 10 (right) and 15 (left) segments remaining; first segment stout, expanded apically, length almost the same as the distance between antennal bases; second segment short, quadrate; third segment expanding apically, widest width almost the same as the length; fourth segment almost quadrate; fifth segment almost quadrate (left side) to 1.5 times longer than the width (right side); remaining segments gradually lengthening. Eyes prominent, slightly shorter than post-ocular length. Pronotum (Fig. 2) slightly longer than broad; anterior margin almost straight; sides parallel; hind margin broadly rounded; median sulcus distinct; prozona weakly raised. Tegmina (Fig. 3) well developed, 1.5 times wider than pronotum, about twice as long as pronotum; broad triangular scutellum visible. Wings (Fig. 3) well-developed. Legs long, slender; hind tarsi with first segment 2.5 times longer than third, second segment about half as long as third, claw with small arolium. Abdomen long, cylindrical; segments eight and nine slightly expanded. Penultimate (= 9^th^) sternite (Fig. 4) slender, with shallow concave sides; caudal margin forming a more or less semicircular lobe. Ultimate (= 10^th^) tergite (Fig. 5) moderately inflated, oval; caudal margin shallow concave between forceps. Forceps (Fig. 5) robust and short, almost straight, pubescent especially on inner margins, tapering and weakly curving inward apically, base of inner margin with small tooth. Genitalia (Figs 6, 7); virga short and sinuated; parameres (= external parameres) short and broad, rounded, with small triangular sclerotized tubercle and sparse short hairs at apex.

***Female.*** Unknown.

######### Remarks.

The subfamily Cylindrogastrinae consists of six species belonging to the Neotropical genus *Cylindrogaster* (Hincks 1955; Steinmann 1986, 1989b). Their external appearance resembles those of the Diplatyinae species or Diplatyidae *sensu stricto* (Table 1). However, males of *Cylindrogaster* spp., including *C. cavernicola* sp. n. described here, have one gonopore on each of the paired virgae, which is a heavily sclerotized process containing the terminal part of the ejaculatory duct, whereas male diplatyids (and haplodiplatyids) have a pair of bifurcated virgae (Hincks 1955; Steinmann 1989a).

**Table 1.** Proposed classification systems for the infraorder Protodermaptera Zacher, 1910.

Hincks (1955, 1959)	Popham (1985)	Steinmann (1986, 1989b)	Sakai (1996)	Engel and Haas (2007)	This study
**Pygidicranidae**	**Pygidicranidae**	**Pygidicranidae**	**Pygidicranidae**	**Pygidicranidae**	**Pygidicranidae**
Anataeliinae	Anataeliinae	Anataeliinae	Anataelinae	Anataeliinae	Anataelinae
Blandicinae	Blandicinae	Blandicinae	Blandicinae	Blandicinae	Blandicinae
		Brindlensiinae	Brindlensiinae	Brindlensiinae	Brindlensiinae
	Chaliinae	Challiinae	Chaliinae	Challiinae	Chaliinae
Echinosomatinae	Echinosomatinae	Echinosomatinae	Echinosomatinae	Echinosomatinae	Echinosomatinae
			Prolabiscinae	(= Prolabiscinae)	Prolabiscinae
Esphalmeninae	Esphalmeninae	Esphalmeninae	Esphalmeninae	Esphalmeninae	Esphalmeninae
Pygidicraninae	Pygidicraninae	Pygidicraninae	Pygidicraninae	Pygidicraninae	Pygidicraninae
Pyragrinae	Pyragrinae	Pyragrinae	Pyragrinae	Pyragrinae	Pyragrinae
				Diplatymorphinae	
				Cylindrogastrinae	
Karschiellinae	Karschiellinae	Karschiellinae	Karschiellinae	**Karschiellidae**	**Karschiellidae**
					**Haplodiplatyidae** (*sensu* Engel et al. 2017)
			**Diplatyidae** (*sensu lato*)	**Diplatyidae** (*sensu stricto*)	**Diplatyidae**
Diplatyinae	Diplatyinae	Diplatyinae	Diplatyinae		Diplatyinae
		Diplatymorphinae	(= Diplatymorphinae)		Diplatymorphinae
(= Cylindrogastrinae)	(= Cylindrogastrinae)	Cylindrogastrinae	Cylindrogastrinae		Cylindrogastrinae

This new species is allied to *C. gracilis* Stal, 1855, which was recorded from Brazil (and also possibly from Peru). However, the virga is entirely straight and the parameres are much wider than the length in the latter species (Fig. 11). Another Brazilian species, *C. thoracicus* Dohrn, 1863, can be distinguished from *C. cavernicola* sp. n. by its much longer virgae (Fig. 10) and pronotum (Fig. 8).

######### Key to the known *Cylindrogaster* species (males only)

**Table:** 

1	Tegmina reduced to small lateral flaps. Ultimate tergite strongly inflated. Forceps well developed, so-called macrolabic	***Cylindrogaster bicyclurus***
–	Tegmina not reduced, well developed, normal. Ultimate tergite not or little inflated. Forceps, so-called microlabic	**2**
2	Parameres of genitalia triangular with pointed apex	**3**
–	Parameres of genitalia trapezoid or oval, broader than long, with blunt apex	**4**
3	Virga short but sinuated, with a characteristic projection at middle	***Cylindrogaster sahlbergi***
–	Virga short, curved but not sinuated, without a characteristic projection at the middle	***Cylindrogaster yepezi***
4	Pronotum (excluding anterior zone tapering to head) apparently longer than broad (Fig. 8)	**5**
–	Pronotum (excluding anterior zone tapering to head) almost quadrate or slightly longer than broad (Figs 2, 9)	**6**
5	Pronotum (excluding anterior tapering region) more than 1.5 times longer than broad. Virga straight, very short, almost half as long as penis lobe	***Cylindrogaster velox***
–	Pronotum (excluding anterior tapering region) less than 1.5 times longer than broad. Virga sinuated, relatively long (Fig. 10)	***Cylindrogaster thoracicus***
6	Virga simple, straight, almost as long as penis lobe (Fig. 11)	***Cylindrogaster gracilis***
–	Virga very short, almost half as long as penis lobe, but weakly sinuated (Figs 6, 7)	***Cylindrogaster cavernicola* Kamimura, sp. n.**

######### Etymology.

The species epithet refers to the cave-dwelling habit of this new species, although it is presently unknown whether it is a troglobite.

######### Distribution.

Rio Grande do Norte, Brazil.

######### Association with caves.

The specimen of *Cylindrogaster cavernicola* Kamimura, sp. n. was collected near the entrance of a cave associated with limestone rocks from the Jandaíra formation (Upper Cretaceous) in northern Rio Grande do Norte state. The caves in this region are predominantly shallow, most of which present several connections with the epigean environment (usually vertical cracks in the limestone outcrops). Accordingly, many caves in the area are strongly influenced by the external environment. Even so, given the extremely dry external environment, the caves represent a more suitable habitat for many animal species, presenting more stable temperatures and higher humidity than the epigean habitat. Furthermore, although the macro-caves are more influenced by the epigean environment, they are connected to huge systems of meso-caves, comprising small passages that are much more stable.

######## Cylindrogaster

Taxon classificationAnimaliaDermapteraPygidicranidae

sp. 1

Figs 12–17

######### Material examined.

1 ♀, Gruta Túneis, Lagoa Santa, Minas Gerais, 10.x.2011, Ferreira, RL leg. (ISLA 43365).

######### Association with caves.

The female specimen was collected near the entrance of a cave of the Sumidouro state park, located in Lagoa Santa, Minas Gerais state. Numerous nymphs presumably belonging to this species were observed throughout the years, especially on the cave walls in areas close to entrances, in the limestone caves of this region. Therefore, it is possible that this species uses the caves as a protected habitat during its development, but leaves the caves when reaching adulthood. They probably prey upon small invertebrates that are found on the walls near the entrances.

######### Description and remarks.

**Figures 12–22.**
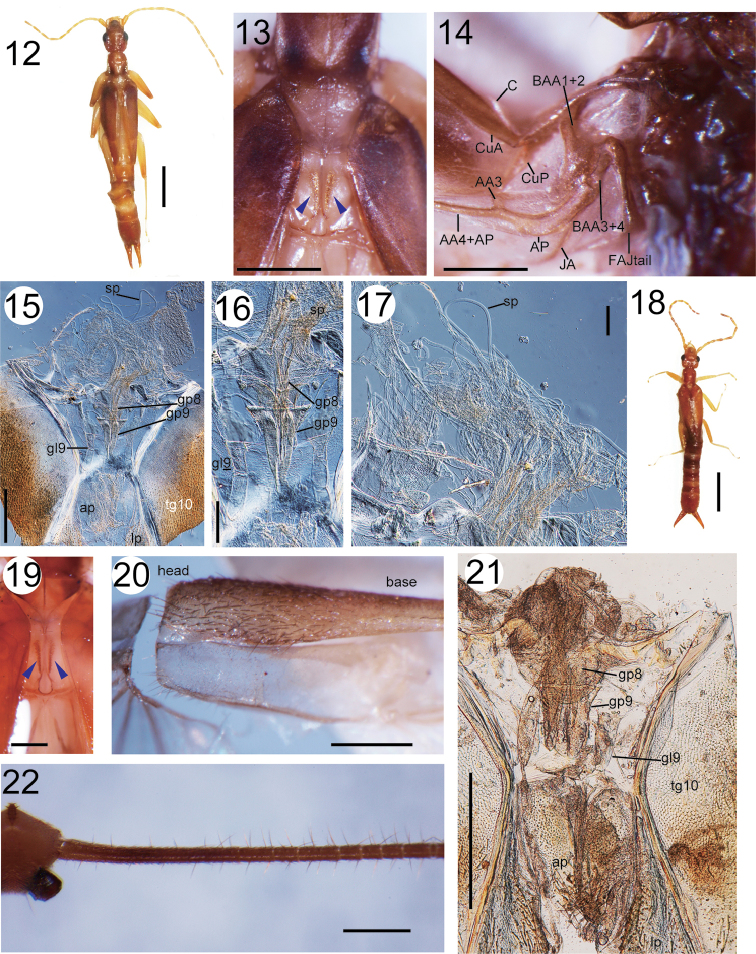
**12–17** *Cylindrogaster* sp. 1 (female): habitus (**12**), spiny ridges (indicated by the blue arrowheads) on the mesothorax (**13**), wing base of the left hindwing (**14**), and genital regions (**15–17**) **18–21** *Cylindrogaster* sp. 2 (female): habitus (**18**), spiny ridges (indicated by the blue arrowheads) on the mesothorax (**19**), fustis of the left hindwing (**20**), and genital regions (**21**) **22** *Cylindrogaster* sp. (nymph): detail of cercus. Scale bars 3 mm for Figs **12, 18**; 0.5 mm for Figs **13–15, 19–22**; 200 μm for Figs **16, 17**.

In this study, two adult females of *Cylindrogaster* were examined. Based on differences in body size and genital structures (see below), these two females are not conspecific. The characteristics for species diagnosis have not been established for female *Cylindrogaster* spp. In addition, because the collection localities of both female samples (from Minas Gerais and Pará states, respectively) are quite far from the type locality of *C. cavernicola* sp. n. (Rio Grande do Norte state), these female samples are tentatively treated as *Cylindrogaster* sp. 1 (Figs 12–17) and *Cylindrogaster* sp. 2 (Figs 18–21) in this study.

Wing and female genital structures have not been reported for any members of *Cylindrogaster*. The female genital region of *Cylindrogaster* sp.1 is characterized by having shorter gonapophysis VIII (**gp8)** than gonapophysis IX (**gp9)**, which was slightly shorter than the finger-like gonoplac IX (**gl9**) (Figs 15–17).

The spermatheca of *Cylindrogaster* sp. 1 consisted of long thin tubes (Fig. 17) without sclerotized or pigmented spermathecal capsules. In the infraorder Protodermaptera (= Pygidicranidae *sensu lato* or sometimes referred to as basal dermapterans), the number of spermatheca and internal branches vary both inter- and intraspecifically (Popham 1965a; Klass 2003; Kamimura 2004). However, poor specimen quality prevented characterization of the *Cylindrogaster* sp. 1 spermatheca.

According to Hass and Kukalová-Peck (2001), Diplatyidae (*Diplatys*) and Haplodiplatyidae (*Haplodiplatys*) are separated from other members of the infraorder Protodermaptera by the presence of (1) a narrow, slender, and elongated fustis head, (2) a long and slender costal area, (3) a concave and strongly three-dimensional anojugal arm (FAJ: anojugal fulcalare), (4) the distal end of CuA3+4 (cubitus anterior 3 and 4) lying between the 8th and 9th branches of AP1+2 (anal posterior 1 and 2), and (5) two proximal branches of AP1+2 diverging close together. The characteristics unique to Diplatyidae and Haplodiplatyidae (1–3) were observed in *Cylindrogaster* sp. 1 (Fig. 14). The latter two traits were not observed as the poor quality of the specimen prevented opening of the wings.

The genus *Haplodiplatys* (Haplodiplatyidae) is characterized by multiple plesiomorphic features, including laterally symmetrical tegmina and absence of a spiny ridge (a component of the tegmina-locking device) on the dorsal side of the mesothorax (Hass and Kukalová-Peck 2001). The female sample of *Cylindrogaster* sp. 1, however, possessed well-developed spiny ridges (Fig. 13), similar to those in *Diplatys* spp. (Diplatyidae).

######## Cylindrogaster

Taxon classificationAnimaliaDermapteraPygidicranidae

sp. 2

Figs 18–21

######### Material examined.

1 ♀, Cave N5SM1-017, Parauapebas, Pará, 17.ii.2011, CARSTE leg. (ISLA 15558).

######### Association with caves.

The specimen was found in the cave N5SM1-017 (also known as GEM-1190 cave). This cave consists of a shallow iron ore cave (14 m horizontal projection), located in Carajás region (Pará state, Brazil). The surrounding vegetation is composed of Amazon forest with a dense canopy. The cave has only one wide and shaded entrance, with the presence of vegetation (lichens, moss, and ferns). Furthermore, it does not present an aphotic zone, being lightened throughout its whole extension. The litter is abundant near the entrance zone and sparse in the rest of the cavity. Several caves were sampled in this region, and as a single specimen was found, it is likely that this species is accidental or seeks only temporarily shelter in caves.

######### Description and remarks.

The genital structures of this species are essentially similar to those of *Cylindrogaster* sp. 1, but the spermatheca was missing likely due to incorrect dissection. *Cylindrogaster* sp. 2 differs from *Cylindrogaster* sp. 1 in having triangular shaped **gl9** (Fig. 21) vs. finger-like in *Cylindrogaster* sp. 1 (Figs 15, 16), as well as a much smaller body size (Fig. 18 vs. 12). The wing and mesothoracic structures of *Cylindrogaster* sp. 2 were basically the same as those of *Cylindrogaster* sp. 1. Refer to Figures 19 and 20 for images of the spiny ridges and fustis, respectively.

######## Cylindrogaster

Taxon classificationAnimaliaDermapteraPygidicranidae

species

Fig. 22

######### Material examined.

1 nymph, Cavidade RF 86, Barão de Cocais, Minas Gerais, 9.ii.2015, ATIVO AMBIENTAL leg. (ISLA 15507) – 1 nymph, Cavidade CBT_09, Barão de Cocais, Minas Gerais, 19.i.2015, ATIVO AMBIENTAL leg. (ISLA 15508) – 1 nymph, S11D, S11D-0003, Serra / Sul, Canaã dos Carajás, Pará, 16.xii.2014, CARSTE leg. (ISLA 17201) – 1 nymph, Gruta da Lapinha, Lagoa Santa, Minas Gerais, 7.vii.2011, Ferreira, RL leg. (ISLA 21083) – 3 nymphs, Cave GEM-1194, Parauapebas, Pará, 23.ii.2011, CARSTE leg. (ISLA 21087) – 1 nymph, CAPA 03, Itabirito, Minas Gerais, 11.xi.2013, SPELAYON leg. (ISLA 21088) – 1 nymph, Cave Mll GEM-1705, Parauapebas, Pará, 13.iv.2011, CARSTE leg. (ISLA 21091) – 2 nymphs, Cave Mll GEM-1738, Parauapebas, Pará, 3.xi.2011, CARSTE leg. (ISLA 21092) – 1 nymph, Cave Mll GEM-1694, Parauapebas, Pará, 20.iii.2011, CARSTE leg. (ISLA 21095) – 4 nymphs, Didi Vieira cave, Afonso Cláudio, Espírito Santo, 23.iii.2005, Souza MS et al. leg. (ISLA 21098) – 1 nymph, Sitio Paraíso cave, Ecoporanga, Espírito Santo, 22.vii.2004, Souza MS et al. leg. (ISLA 21099) – 2 nymphs, Gruta do Roxo, Novo Oriente de Minas, Minas Gerais, 20.vii.2002, Souza MS et al. leg. (ISLA 21100) – 1 nymph, SEP-0407 (Geraldo Gusso), Felipe Guerra, Rio Grande do Norte, 2.x.2010, Ferreira, RL leg. (ISLA 21102) – 1 nymph, Lapa de Urtiga, Vazante, Minas Gerais, 16.ix.2010, Ferreira, RL leg. (ISLA 2735).

**Remarks.** A total of 21 nymphs of *Cylindrogaster* were examined. Nymphal cerci were frequently lost in these specimens, but when present, they were always segmented instead of unsegmented forceps of adults (Fig. 22).

Species diagnosis has not been established for nymphal samples of *Cylindrogaster* spp.

#### Infraorder EPIDERMAPTERA Engel, 2003

##### Family ANISOLABIDIDAE Verhoeff, 1902

###### Subfamily ANISOLABIDINAE Verhoeff, 1902

####### *Euborellia* Burr, 1910

######## Euborellia janeirensis

Taxon classificationAnimaliaDermapteraAnisolabididae

(Dohrn, 1864)

Figs 23–28

######### Material examined.

3 ♂♂, 6 ♀♀, 7 nymphs, Gruta dos Farias cave, Barbalhas, Ceará, 30.iv.2007, Ferreira, RL leg. (ISLA 15565) – 1 nymph, Cave GEM-1623, Parauapebas, Pará, 16.iii.2011, CARSTE leg. (ISLA 21085) – 1 nymph, Gruta Ecos cave, Cocalzinho de Goiás, Goiás, 4.iv.2006, CECAV leg. (ISLA 21096).

######### Association with caves.

While most earwigs found in Brazilian caves seem to be accidental, this species was present as a large population within Gruta dos Farias cave, a sandstone cave located in Barbalhas municipality (Ceará state, Brazil). Many adults and nymphs were observed only in guano piles in deeper areas of the cave, which has a stream trespassing its entire conduit, strongly suggesting that the population is troglophilic. They are likely feeding on bat guano or preying upon small invertebrates.

######### Description and remarks.

**Figures 23–35.**
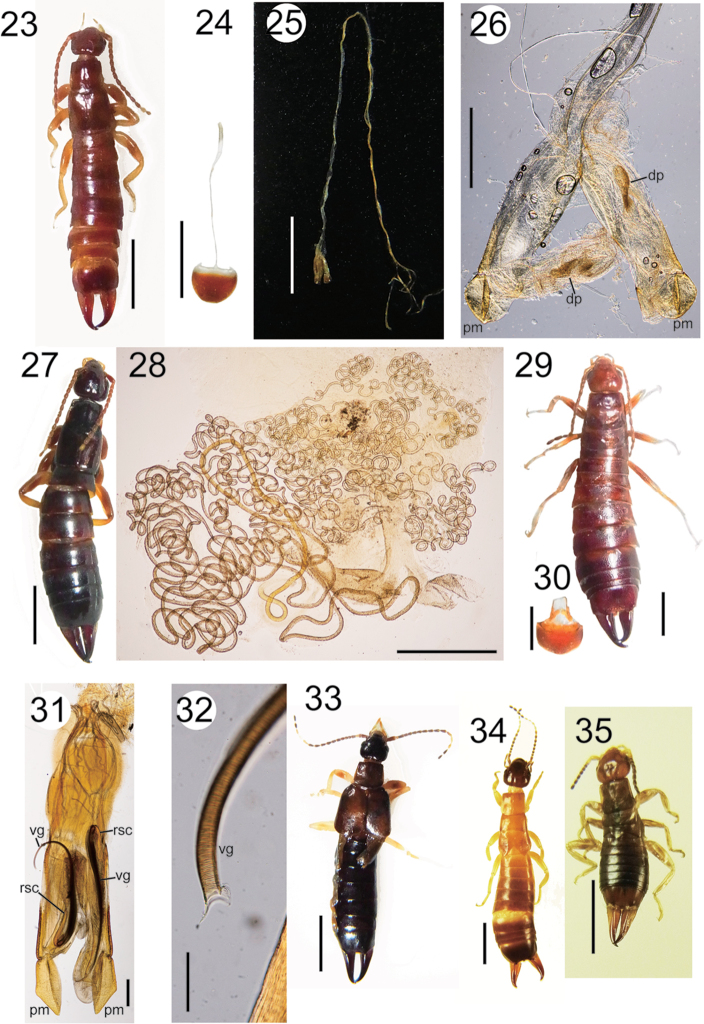
**23–26** *Euborellia janeirensis* (male): habitus (**23**), penultimate sternite + manubrium (**24**), and genitalia (**25, 26**) **27–28** *Euborellia janeirensis* (female): habitus (**27**), and spermatheca (**28**) **29–32** *Euborellia brasiliensis* (male): habitus (**29**), penultimate sternite + manubrium (**30**), and genitalia (**31, 32**) **33** Anisolabididae gen. sp. 1 (female): habitus **34** Anisolabididae gen. sp. 2 (nymph): habitus **35** Anisolabididae gen. sp. 3 (nymph) habitus. Scale bars 3 mm for Figs **23–25, 27, 29, 30, 33–35**; 0.5 mm for Figs **26, 28, 31**; 100 μm for Fig. **32**.

All adult specimens examined in this study had fully developed tegmina, but lacked hind wings. Four species of *Euborellia* from the Neotropical region, *E. boliviana* Brindle, 1971, *E. ambigua* (Borelli, 1906), *E. caraibea* Hebard, 1921, and *E. janeirensis*, also have such characteristics. Among these species, *E. janeirensis* is distinguished from the others by the presence of well-developed lateral longitudinal ridges on the male abdominal tergites VI and IX, and one or more white/yellow distal antennal segments (Steinmann 1989a). The external morphologies and male genitalia of the specimens examined in this study agreed well with those described previously for *E. janeirensis*, including brown markings on the femora (Figs 23 and 27), the shapes of the forceps (Figs 23 and 27), and the shapes of the parameres and denticulated pads in the penis lobe (Fig. 26).

Taxonomists generally examine only the terminal region of the male genital organs. The male genitalia of *E. janeirensis* were approximately 22 mm in length, and more than twice the body length with forceps (Fig. 25). The manubrium, which is an extension in the basal, inner margin of the penultimate sternite (Burr 1915b; Ramamurthi 1958), was approximately 6 mm in length (Fig. 24). In male earwigs, the retractor muscles of the genitalia originate from this structure (Popham 1965a). As reported by Mariani (1994), the spermatheca of this species was a long and thin blind duct lacking a capsule at the distal end (Fig. 28).

Males of *Euborellia* spp. directly insert the elongated virga into the female spermatheca during copulation (Kamimura 2000; Lieshout and Elgar 2011). Elongation of the virga (and the genitalia as a whole, which functions as the virgal sheath when in repose) is considered an adaptation for removal of rival sperm from the female spermatheca, which is usually longer than the virga (Kamimura 2000, 2005, 2013, 2015; Lieshout and Elgar 2011). Thus, genital elongation in *E. janeirensis* suggests intensive sperm competition in this species.

######### Distribution.

Brazil, Argentina, Paraguay, and Venezuela.

######## Euborellia brasiliensis

Taxon classificationAnimaliaDermapteraAnisolabididae

(Borelli, 1912)

Figs 29–32

######### Material examined.

2 ♂♂, 2 nymphs, Gruta dos Coelhos cave, Lima Duarte, Minas Gerais, 11.vii.2005, Ferreira, RL leg. (ISLA 15564).

######### Association with caves.

This species was found in deeper areas (aphotic zones) of Gruta dos Coelhos and Gruta do Pião caves, both associated with quartzite rocks and located in the Ibitipoca state park (Lima Duarte municipality, Minas Gerais state, Brazil). In the latter cave, several individuals were found walking near root masses, probably searching for prey. There are several caves in the area, and at least three distinct inventories of cave fauna were performed over the last 10 years. However, this species was only found in two caves, suggesting that, although probably not accidental, their association with subterranean habitats is uncommon.

######### Remarks.

This apterous anisolabidid species from Brazil (Fig. 29) was originally described as *Heterolabis brasiliensis* by Borelli (1912), as the type species of the monotypic genus *Heterolabis*. Popham and Brindle (1966) recognized the type species as a member of the genus *Euborellia* Burr, 1910 and proposed the combination *Euborellia brasiliensis* (Borelli, 1912) Popham & Brindle, 1966. Therefore, they considered the generic name *Heterolabis* as a synonym of *Euborellia*. Their decision was followed by Reichardt (1968b), Sakai (1970), and Steinmann (1977, 1978).

Srivastava (1978) described a second species belonging to *Heterolabis*, i.e. *Heterolabis punctata* Srivastava, 1978. Its transfer to the genus *Euborellia* was proposed by Sakai (1982, 1987) and Srivastava (1999). Since the binomen *Euborellia punctata* was preoccupied by *Euborellia punctata* Borelli, 1927, they proposed the replacement names *Euborellia srivastavai* Sakai, 1987 and *E. mindanoensis* Srivastava, 1999, respectively. An alternative generic position for *H. punctata* was proposed by Steinmann (1989b); who recognized it as a member of the genus *Epilabis* Burr, 1915. Since the relevant binomen was preoccupied by *Epilabis punctata* Srivastava, 1976, he proposed the replacement name *Epilabis harlequin* Steinmann, 1989.

Currently *Heterolabis* Borelli, 1912 is considered a synonym of *Euborellia*, and the species previously considered as members of *Heterolabis* is as follows:

1. *Euborellia brasiliensis* (Borelli, 1912): Popham and Brindle (1966)

Synonym: *Heterolabis brasiliensis* Borelli, 1912

2. *Euborellia srivastavai* Sakai, 1987

Synonyms: *Heterolabis punctata* Srivastava, 1978; *Epilabis harlequin* Steinmann, 1989 (junior objective synonym); *Euborellia mindanoensis* Srivastava, 1999 (junior objective synonym).

The generic name *Heterolabis* Borelli, 1912 is an invalid homonym of *Heterolabis* Kriechbaumer, 1889 (Ichneumonidae).

######### Distribution.

Brazil.

####### Anisolabidinae

Taxon classificationAnimaliaDermapteraAnisolabididae

sp. 1

Fig. 33

######## Material examined.

1 ♀, cave SERP 0100, Conceição do Mato Dentro, Minas Gerais, 26.v.2014, SPELAYON leg. (ISLA 15557).

######## Association with caves.

Unknown.

######## Remarks.

The tegmina and wings were fully developed (Fig. 33), and this species may belong to the genus *Carcinophora* Scudder, 1876.

####### Anisolabidinae

Taxon classificationAnimaliaDermapteraAnisolabididae

sp. 2

Fig. 34

######## Material examined.

2 nymphs, Gruta Ecos cave, Cocalzinho de Goiás, Goiás, 4.iv.2006, CECAV leg. (ISLA 21097) – 1 nymph, Cave GEM-1710, Parauapebas, Pará, 14.iii.2011, CARSTE leg. (ISLA 21086) – 1 nymph, cave SERP 0100, Conceição do Mato Dentro, Minas Gerais, 26.v.2014, SPELAYON leg. (ISLA 21082).

######## Association with caves.

Unknown.

######## Remarks.

Four nymphs, with well-developed wing primordia and some whitish antennal segments, were examined. Being recorded from Goiás, Pará, and Minas Gerais states, this species is possibly distributed widely in Brazil. Otherwise, several species may be mixed in this tentative species.

####### Anisolabidinae

Taxon classificationAnimaliaDermapteraAnisolabididae

sp. 3

Fig. 35

######## Material examined.

1 nymph, cave RF 103, Barão de Cocais, Minas Gerais, 16.i.2015, ATIVO AMBIENTAL leg. (ISLA 15505).

######## Association with caves.

Unknown.

######## Remarks.

Only one young nymphal specimen was collected. This species is characterized by a brownish stripe straddling the long axis of the compound eye.

##### Family SPONGIPHORIDAE Verhoeff, 1902

###### Spongiphoridae

Taxon classificationAnimaliaDermapteraSpongiphoridae

sp. 1

####### Material examined.

1 nymph, Cave Mll GEM 1712, Parauapebas, Pará, 30.x.2011, CARSTE leg. (ISLA 21093).

####### Association with caves.

Unknown.

####### Remarks.

This species is tentatively assigned to the family Spongiphoridae. Only one young nymph, which lacked the post-abdomen including the forceps, was examined. The antennae are characteristically stout.

###### Subfamily LABIINAE Burr, 1911

####### *Paralabellula* Kevan, 1997

(= *Paralabella* Steinmann, 1990; see Kevan and Vickery 1997)

######## Paralabellula dorsalis

Taxon classificationAnimaliaDermapteraFlabellulidae

(Burmeister, 1838)

Figs 36–42

######### Material examined.

2 ♂♂, 9 ♀♀, 8 nymphs, Cave N5SM2-099 (= GEM-1799 cave), Parauapebas, Pará, 5.v.2011, CARSTE leg. (ISLA 15563) – 1 ♀, Cave N5SM2-019 (= GEM-1739 cave), Parauapebas, Pará, 31.x.2010, CARSTE leg. (ISLA 15562) – 3♀, Cave N5SM2-099 (= GEM-1799 cave), Parauapebas, Pará, 31.x.2010, CARSTE leg. (ISLA 15560) – 1 ♂, 2 ♀♀, 8 nymphs, Cave N5SM2-019 (= GEM-1739 cave), Parauapebas, Pará, 5.v.2011, CARSTE leg. (ISLA 15561) – 1 nymph, Cave N5SM2-019 (= GEM-1739 cave), Parauapebas, Pará, 31.x.2010, CARSTE leg. (ISLA 21084) – 1 nymph, Cave N5SM2-019 (= GEM-1739 cave), Parauapebas, Pará, 5.v.2011, CARSTE leg. (ISLA 21089) – 1 nymph, Cave N5SM2-019 (= GEM-1739 cave), Parauapebas, Pará, 5.v.2011, CARSTE leg. (ISLA 21090) – 3 nymphs, Cave N5SM2-019 (= GEM-1739 cave), Parauapebas, Pará, 31.x.2010, CARSTE leg. (ISLA 21094).

######### Association with caves.

Many specimens of *P. dorsalis* were found in two iron ore caves (N5SM2-019 cave – synonym of GEM-1739 cave, and N5SM2-099 cave – synonym of GEM-1799 cave), both located in Carajás region (Pará state). These caves occur in an iron ore plateau surrounded by the Amazon forest. However, they are in an area of metallophilic savannah, a vegetation type usually found at the top of plateaus. The caves are considerably large (> 100 m in horizontal projection) compared to other caves in the area. Although many caves were sampled in the plateau (at least 100 caves), this species was found in only these two caves, which contain huge colonies of the insectivorous bat genus *Pteronotus* (*Pteronotus gymnonotus* Wagner, 1843, in the N5SM2-019 cave and *Pteronotus parnellii* (Gray, 1843) in the N5SM2-099 cave). These colonies produce large guano piles, where several individuals of *P. dorsalis* were observed. The populations of *P. dorsalis*, which include both adults and nymphs, were observed in both dry and rainy seasons in the two caves, strongly suggesting that they are troglophilic.

######### Description and remarks.

**Figures 36–45.**
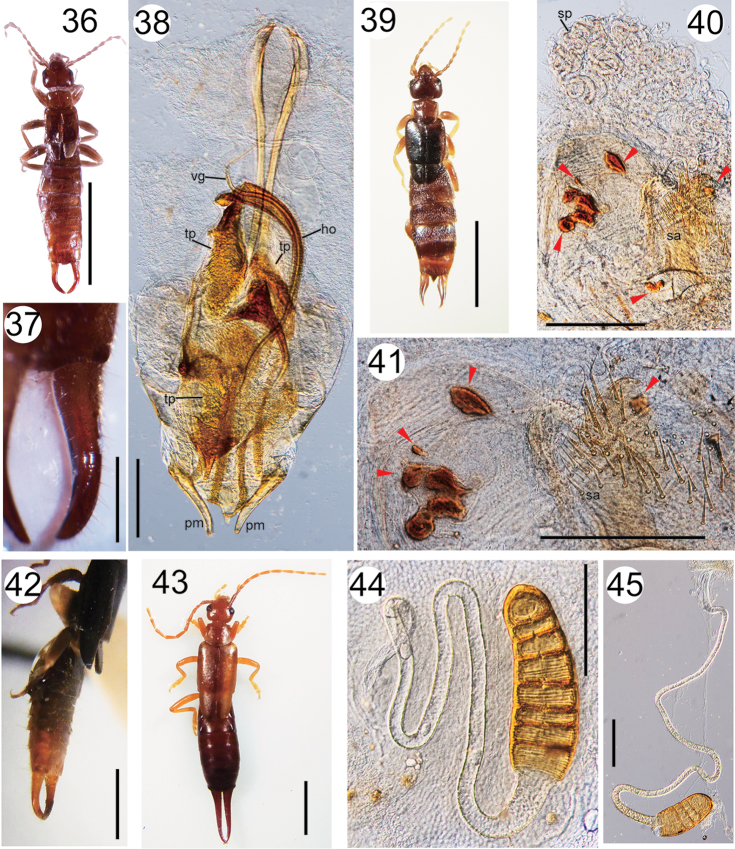
**36–38** *Paralabellula dorsalis* (male): habitus (**36**), forceps (dorsolateral view) (**37**), and genitalia (**38**) **39–41** *Paralabellula dorsalis* (female): habitus (**39**), spermatheca and spiny area in the genitalia (**40, 41**). Repaired wound patches are indicated by the red arrowheads in Figs 40 and 41 **42** *Paralabellula dorsalis* (male; a specimen from Colombia, det. A. Brindle; OMHH S. Sakai Collection, 31.iii.2002 [01-25]): abdomen and forceps (dorsolateral view) **43–44** *Doru luteipes* (female): habitus (**43**), and spermatheca (**44**) **45** *Doru lineare* (female; coll. Y. Kamimura, at Varzelândia, Minas Gerais, Brazil, outside of caves on 13.iii.2016): spermatheca. Scale bars 3 mm for Figs **36, 39, 42, 43**; 0.5 mm for **37**; 200 μm for Figs **38, 40, 41, 44, 45**.

The external morphologies and genital structures of male and female specimens were examined (Figs 36–41) and matched those of *Paralabellula dorsalis* (Burmeister 1838; Brunner 1906; Brindle 1971a,b,c; Steinmann 1989a; Briceño 1997; Eberhard et al. 1998). Winged and wingless morphs have been reported for a Costa Rican population of this species, where wingless individuals always predominated in the wild (Briceño and Eberhard 1987). Significantly more winged adults emerged when nymphs were subjected to low nutritional conditions (Briceño and Eberhard 1987). All adults (3 males and 13 females) examined in this study were winged morphs with fully developed tegmina and hind wings (Figs 36, 39). These findings may have been due to the poor nutritional conditions in caves. Although previous authors noted that each branch of the male forceps has a small inner tooth at the base (see Brindle 1971a, b, c; Sakai 1993), all males (n = 3) examined in this study lacked this tooth (Fig. 37). However, one male specimen from Moniquira, Colombia, determined by A. Brindle, also lacked a tooth at the base of the forceps (Fig. 42).

Based on samples collected from Costa Rica, Briceño (1997) reported the detailed genital structures of this species, which included a horn-like structure and several heavily sclerotized toothed plates in the penis lobe. These structures were also found in the three male samples examined in this study (**ho** and **tp**, respectively, in Fig. 38). The spermatheca of this species is a long, thin, blind duct lacking a capsule at the distal end (Mariani 1994; Briceño 1997). Briceño (1997) also reported the presence of multiple fine spines around the spermathecal opening. All such features were present in the specimens examined in this study (Fig. 40, 41).

Using specimens fixed during copulation, Briceño (1997) also examined coupling of the male and female genitalia. Toothed plates were exposed by eversion of the inflated penis lobe, and contacted the inner walls of the vagina, including the spiny area. The horn-like structure functioned as a guiding sheath for the virga, potentially to facilitate insertion into the female spermatheca. There is accumulating evidence that many male animals inflict wounds on the female during mating through use of their genital structures (Lange et al. 2013; Tatarnic et al. 2014; Reinhardt et al. 2014). However, this mode of mating, termed traumatic mating, has been reported in only two species of earwigs, *Echinosoma denticulatum* Hincks, 1959 (Pygidicranidae: Echinosomatinae; Kamimura and Lee 2014a) and *Marava arachidis* (Yersin, 1860) (Spongiphoridae: Spongiphorinae; Kamimura et al. 2016b). While Briceño (1997) failed to mention the occurrence of copulatory wounding in *P. dorsalis*, melanized patches on the membranous region at the spermathecal opening were observed in this study (n = 2; Figs 40, 41). This finding suggests that the male genitalia cause wounding during copulation.

######### Distribution.

West Indies, Mexico, Costa Rica, Panama and northern South America (including Brazil).

##### Family FORFICULIDAE Latreille, 1810

###### Subfamily FORFICULINAE Latreille, 1810

####### *Doru* Burr, 1907

######## Doru luteipes

Taxon classificationAnimaliaDermapteraForficulidae

(Scudder, 1876)

Figs 43 , 44

######### Material examined.

1 ♀, Gruta do Vento cave, Pains, Minas Gerais, 12.x.2000, R. L. Ferreira leg. (ISLA 495) – 1 ♀, Gruta Zé Geraldão cave, Pains, Minas Gerais, 10.iii.2009, R. A. Zampaulo leg. (ISLA 534).

######### Association with caves.

*Doru luteipes* is an extremely common species in Brazil (Reis et al. 1988), being frequently associated with crops, especially corn. The few observed specimens were found from the Pains region, which is considered the speleological area with the highest concentration of caves in Brazil (and probably in South America). Although more than 300 caves have been examined in this area (Zampaulo 2010), this species was found near the entrances of only two caves, suggesting that they are definitely accidental.

######### Remarks.

Only female samples of *Doru*, which are difficult to identify to the species level, were collected in this study. The external morphologies (Fig. 43) matched those of *D. luteipes* (Scudder 1876; Brindle 1971d; Steinmann 1979; 1993). The females of *D. luteipes* are similar in appearance to *Doru lineare* (Eschscholtz, 1822) [Brindle 1971d; Steinmann 1979, 1993: see also Sakai 1993, 1995 for proposal of a synonymy of this species with *Doru taeniatum* (Dohrn, 1862)]; however, the spermathecal morphologies, including the shape of the spermathecal capsule with seven constrictions (Fig. 44: the spermathecal morphology was examined for the sample from Gruta do Vento cave), were the same as those described by Mariani (1994) for *D. luteipes*. According to Mariani (1994), the spermathecal capsule of *D. lineare* is shorter with fewer and weaker constrictions. This was confirmed in a female sample collected at Varzelândia, Minas Gerais, Brazil on 13.iii.2016 by Y.K., with conspecific males (Fig. 45). However, parthenogenesis has been reported for some Brazilian populations of *D. lineare* (Cocco et al. 2013), suggesting possible polymorphisms in the spermathecal morphology. Thus, the identification of the samples is tentative.

######### Distribution.

Colombia, Surinam, Brazil, Peru, Bolivia, and Argentina.

## Discussion

Of the five taxa identified to the species level in this study, *E. brasiliensis* and *D. luteipes* may be accidental inhabitants of caves, while *E. janeirensis* and *P. dorsalis* likely maintain permanent cave populations, but being also found in various non-cave habitats (troglophiles).

At present the association to cave habitats is unknown for *C. cavernicola* sp. n. The species does not present any obvious troglomorphic traits. However, subterranean species do not always possess obvious troglomorphic traits, especially when associated with shallow subterranean habitats. Bartkowiak et al. (1991) compared the eyes of carabids with different degrees of adaptation for cave life, and suggested that troglobites living in superficial subterranean systems are able to use dim light stimuli for orientation using eyes. Some troglobitic carabids that live in shallow subterranean habitats in the Amazon with only partially reduced eyes also support this view (Pellegrini and Ferreira 2011). Accordingly, if *C. cavernicola* sp. n. is mainly associated with shallow subterranean habitats (such as the caves in the type locality) in the extremely dry area with high insolation, it could maintain functional eyes. Further samples and studies on the ecology of this new species are necessary, before determining whether caves are its primary habitat.

The association of Dermaptera with bat guano in caves has been reported for some species around the world: large populations of *Arixenia esau* Jordan, 1909 associated with bat guano piles in Niah caves, Sarawak (Medway 1958), and *Xeniaria* species occurring in high densities in bat guano of caves in the Gunong Mulu National Park (Borneo) (Brindle 1980b). Askew (1971) also reported *Xeniaria jacobsoni* (Burr, 1912) occurring in high densities in bat guano piles in a cave in Java where they prey upon small arthropods. In addition to these Arixeniids, many other species observed in caves were also collected near guano piles (*Irdex chapmani* Brindle, 1980 and *Nala ornata* Borelli, 1932 from caves in Borneo; *Schizochelisoches brevipennis* from caves in Peninsular Malaysia) (Brindle and Oromi 1994), suggesting that guano is an important direct or indirect resource for earwigs in caves. However, except for *E. janeirensis* and *P. dorsalis*, most species found in caves in Brazil seem to be accidental or not directly associated with guano. The reasons for this lack of association remain unknown. Additional studies are necessary to confirm the relations of Brazilian cave-dwelling earwigs to cave environments, as in most cases, only one or two samples were obtained in each cave.

### Systematic position of the subfamily Cylindrogastrinae

In the present study, the wing and female genital structures are described for *Cylindrogaster* spp. for the first time. Well-developed ovipositor components [gonoplacs (= coxal lobes) and gonapophyses of the 8th and 9th abdominal segments (gl8, gl9, gp8, and gp9, respectively)] are considered plesiomorphic in Dermaptera (Zacher 1911; Burr 1915a; Hincks 1959; Giles 1963; Klass 2003).

Klass (2003) studied the female genitalia of almost all representative groups of the Protodermaptera (Karschellidae, Haplodiplatyidae, Diplatyidae, and Pygidicranidae *sensu stricto*). Many members of Pygidicranidae *sensu stricto*, including *Echinosoma* (Echinosomatinae), *Dacnodes* (Pygidicraninae), *Tagalina* (Pygidicraninae), and *Anataelia* (Anataelinae), are characterized by a well-developed pair of slender gp8, and a pair of long lobate gl9 that are longer than gl8 and gp9 (Klass 2003). Conversely, the gp8 in the female genitalia of Diplatyinae (*Diplatys*) and Haplodiplatyidae (*Haplodiplatys*) is reduced and usually shorter than gl9 (Klass 2003; Kamimura et al. 2016a). This characteristic is shared with *Karschiella* (Karshiellidae) and *Esphalmenus* (Pygidicranidae *sensu stricto*, Esphalmeninae) (Klass 2003). Thus, the female genitalia of *Cylindrogaster* spp. are of the Diplatyinae ‒ Karshiellidae ‒ *Esphalmenus* type.

Klass (2003) also found a large unpaired gland in nearly all species. A pair of thin, long cuticular tubes (referred to as lateral tubes in Klass 2003) are associated with this gland, which opens on the midline of the body on segment IX. The only exception to this finding was *Diplatys* (Klass 2003). Klass (2003) noted that no similar organs have been recorded in other insect taxa, and thus the lateral tubes are likely autapomorphic for Dermaptera or for a subgroup of them. Lateral tubes have also been reported for *Esphalmenus* (Pygidicranidae) and *Allostethus* (Labiduridae *sensu lato*), which lack the unpaired gland (Klass 2003; Kamimura and Lee 2014b). In this study, we failed to detect an unpaired gland and lateral tubes in the female genital region of *Cylindrogaster* sp. 1 and *Cylindrogaster* sp. 2. Among the members of Protodermaptera, a similar observation was reported for *Diplatys*, in which only a small fold was present immediately behind the base of gonapophyses IX. It was hypothesized that the fold was a vestige of the accessory gland or of the common entrance pouch of the lateral tubes (Klass 2003).

In conclusion, multiple genital and wing traits (see “Remarks” of *Cylindrogaster* sp. 1 and *Cylindrogaster* sp. 2), as well as segmented nymphal cerci, suggested a close relationship between Cylindrogastrinae and Diplatyinae (or Diplatyidae *sensu stricto*). These two groups in the Protodermaptera also had depressed femora with lamelliform edges, an arolium between the claws, and short antennae with fewer than 25 segments (Hincks 1955, 1959; Steinmann 1986). The most notable difference between the two groups is the number of gonopores per virga. However, bifurcated virgae have also been reported in other groups of Pygidicranidae, including the genera *Pyragra*, *Esphalmenus*, and *Cranopygia* (Hincks 1959; Popham 1965a; Brindle 1984).

The subfamily Cylindrogastrinae was originally erected in the family Pygidicranidae (Maccagno 1929). The subfamily was not recognized by Hincks (1955), Popham (1965b, 1969, 1985), Reichardt (1968a) and Günther and Herter (1974) due to its similar external morphology to that of Diplatyinae (Tab. 1). However, Sakai (1971, 1982, 1985, 1996) considered the differences in the virgal morphologies (1 vs. 2 gonopores) important and recognized Cylindrogastrinae and Diplatyinae as distinct subfamilies within Diplatyidae. Steinmann (1975, 1986, 1989b) proposed Diplatyinae and Cylindrogastrinae as subfamilies of the family Pygidicranidae (Tab. 1). Differently, some subsequent studies placed Cylindrogastrinae within Pygidicranidae, while treating Diplatyidae at the family level (Haas and Klass 2003; Engel and Haas 2007). We support the position of Cylindrogastrinae in the Diplatyidae and not in Pygidicranidae (Tab. 1). The subfamily Diplatymorphinae includes only one species, *Diplatymorpha borneensis* Boeseman, 1954, which was described based on a single female specimen in Borneo (Boeseman 1954). According to Steinmann (1986), this subfamily can be separated from the Cylindrogastrinae based on the shape of fifth antennal segment: almost quadrate as the fourth segment in *Diplatymorpha borneensis* whereas visibly longer than the fourth segment in *Cylindrogaster* spp. However, as described for the holotype of *Cylindrogaster cavernicola* sp. n., there may be a considerable variation in the antennal morphology even within an individual. Except for the antennal morphology and slightly sturdier built body, the external morphology of *Diplatymorpha borneensis* resembles those of *Diplatys* species (Boeseman 1954), and therefore multiple researchers treat it as a junior synonym of Diplatyinae (Tab. 1). Accordingly, we tentatively place Diplatymorphinae as a distinct subfamily within Diplatyidae, while Diplatyidae, Haplodiplatyidae, and Pygidicranidae are treated as separate families.

## Supplementary Material

XML Treatment for Cylindrogaster cavernicola

XML Treatment for Cylindrogaster

XML Treatment for Cylindrogaster

XML Treatment for Cylindrogaster

XML Treatment for Euborellia janeirensis

XML Treatment for Euborellia brasiliensis

XML Treatment for Anisolabidinae

XML Treatment for Anisolabidinae

XML Treatment for Anisolabidinae

XML Treatment for Spongiphoridae

XML Treatment for Paralabellula dorsalis

XML Treatment for Doru luteipes
